# Developing an Intervention for Fall-Related Injuries in Dementia (DIFRID): an integrated, mixed-methods approach

**DOI:** 10.1186/s12877-019-1066-6

**Published:** 2019-02-28

**Authors:** Alison Wheatley, Claire Bamford, Caroline Shaw, Elizabeth Flynn, Amy Smith, Fiona Beyer, Chris Fox, Robert Barber, Steve W. Parry, Denise Howel, Tara Homer, Louise Robinson, Louise M. Allan

**Affiliations:** 1 Institute of Health and Society, Newcastle University, Newcastle upon Tyne, England; 2 The Newcastle upon Tyne Hospitals NHS Foundation Trust, Newcastle upon Tyne, England; 3 Tees, Esk and Wear Valleys NHS Foundation Trust, Darlington, England; 4 University of East Anglia, Norwich, England; 5 Northumberland Tyne and Wear NHS Foundation Trust, Newcastle upon Tyne, England; 6 Newcastle University, Newcastle upon Tyne, England; 7 Institute of Health Research, University of Exeter, South Cloisters, St Luke’s Campus, Heavitree Road, Exeter, EX1 2LU England

**Keywords:** Dementia, Falls, Intervention development, Delphi consensus, Realist synthesis

## Abstract

**Background:**

Falls in people with dementia can result in a number of physical and psychosocial consequences. However, there is limited evidence to inform how best to deliver services to people with dementia following a fall. The aim of the DIFRID study was to determine the feasibility of developing and implementing a new intervention to improve outcomes for people with dementia with fall-related injuries; this encompasses both short-term recovery and reducing the likelihood of future falls. This paper details the development of the DIFRID intervention.

**Methods:**

The intervention was designed using an integrated, mixed-methods approach. This involved a realist synthesis of the literature and qualitative data gathered through interviews and focus groups with health and social care professionals (*n* = 81). An effectiveness review and further interviews and observation were also conducted and are reported elsewhere. A modified Delphi panel approach with 24 experts was then used to establish a consensus on how the findings should translate into a new intervention. After feedback from key stakeholders (*n* = 15) on the proposed model, the intervention was manualised and training developed.

**Results:**

We identified key components of a new intervention covering three broad areas:

• Ensuring that the circumstances of rehabilitation are optimised for people with dementia

• Compensating for the reduced ability of people with dementia to self-manage

• Equipping the workforce with the necessary skills and information to care for this patient group

Consensus was achieved on 54 of 69 statements over two rounds of the Delphi surveys. The statements were used to model the intervention and finalise the accompanying manual and protocol for a feasibility study. Stakeholder feedback was generally positive and the majority of suggested intervention components were approved. The proposed outcome was a 12-week complex multidisciplinary intervention primarily based at the patient’s home.

**Conclusions:**

A new intervention has been developed to improve outcomes for people with dementia following a fall requiring healthcare attention. The feasibility of this intervention is currently being tested.

**Trial registration:**

ISRCTN41760734 (16/11/2015).

**Electronic supplementary material:**

The online version of this article (10.1186/s12877-019-1066-6) contains supplementary material, which is available to authorized users.

## Background

People with dementia who live in their own home make up 70% of all people living with dementia in the UK [1], and are ten times more like to fall as people without dementia [2]. The negative consequences of falls are greater for people with dementia than for other older people [3]. While even non-injurious falls can result in psychosocial consequences such as loss of confidence and fear of falling [4], functional decline in people who sustain injuries may be greater than in those who do not sustain injuries [5, 6]. Despite this, few trials have specifically addressed the management of fall-related injuries in people with dementia. While multifactorial interventions by specialist falls services are effective in preventing further falls in older people without dementia [7, 8], evidence of their effectiveness for people with dementia is inconclusive [9–11]. Similarly, falls-prevention exercise programmes such as Otago [12] have little evidence of efficacy for people with dementia, though some work has been done on tailoring the programme for individuals [13, 14]. There is, however, some evidence that rehabilitation interventions may result in improvements in motor performance in people with dementia [15] and that motor training can increase physical activity in people with dementia without increasing the risk of falls [16]. Recently published guidelines acknowledge that multifactorial falls interventions may not be suitable for a person living with severe dementia, but provide no recommendations on how to optimise falls interventions for this patient group [17].

The brief for this study was therefore to develop a new complex intervention to improve care for community-dwelling people with dementia with fall-related injuries. In response to calls for a more systematic approach to, and greater transparency in, intervention development [18–20], this paper describes the development process in detail. This includes presenting (a) the causal factors and change mechanisms underpinning falls and rehabilitation care for this patient group; (b) the outcomes of a consensus-seeking process based on this initial work; (c) the development of a logic model; and (d) the development of intervention materials.

## Methods

**Fig. 1:**
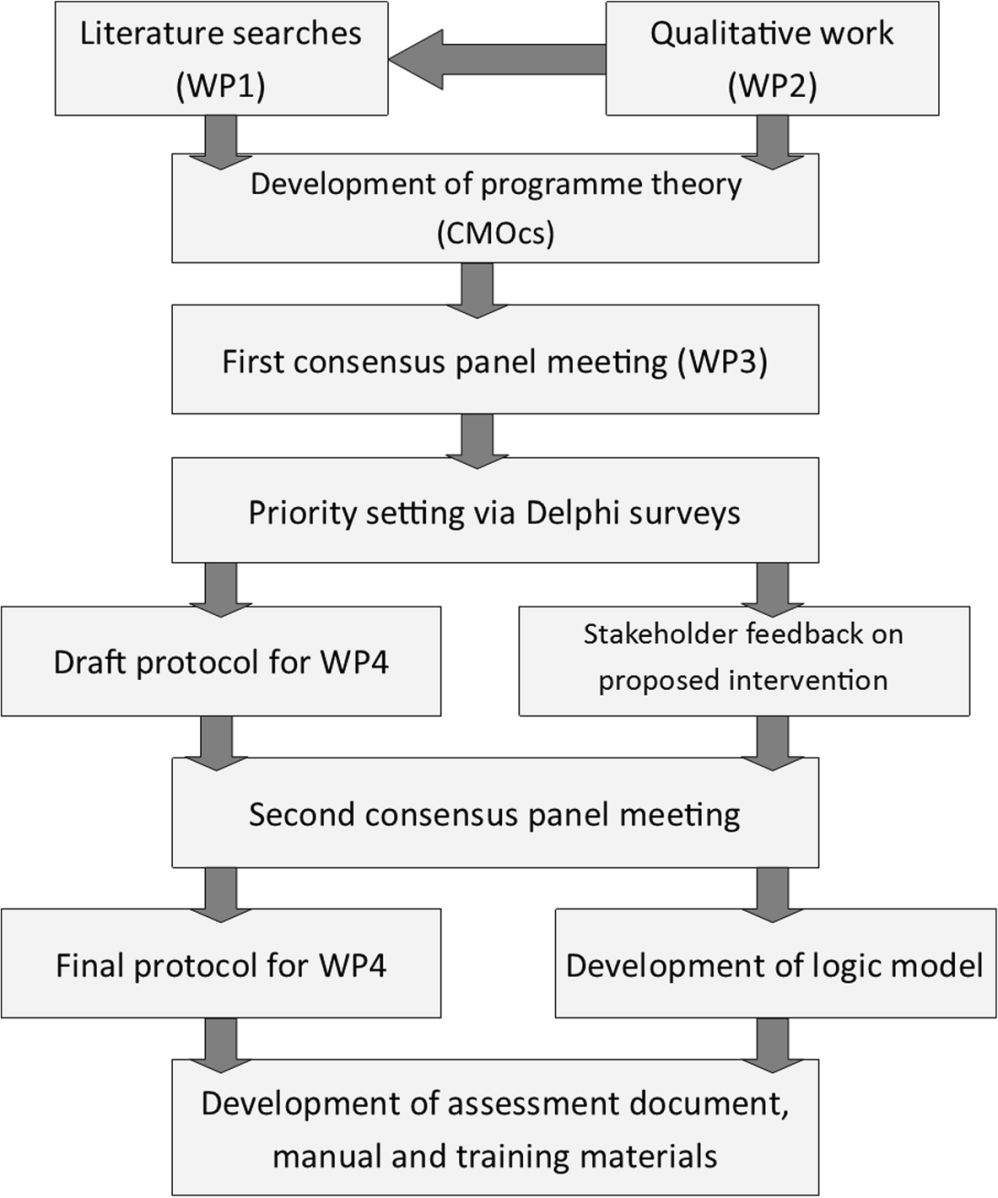
Intervention development

The development of the intervention involved qualitative work to map existing care pathways [21] and explore the views of stakeholders on the content and delivery of a new intervention [22]; an effectiveness review [5]; a realist synthesis of the literature; a prospective diary study to provide information on recruitment sources and existing service use; consensus panel meetings of experts; a Delphi survey; and further qualitative work to elicit stakeholder feedback on the proposed intervention. The findings were then used to develop a logic model, protocol [23] and intervention materials. The feasibility and acceptability of the new intervention is currently being evaluated. Figure 1 illustrates the process of intervention development.

### Identifying causal factors and change mechanisms

#### Qualitative work and formative realist analysis

The initial qualitative work comprised 58 semi-structured interviews and 5 focus groups with health and social care professionals (full details of qualitative work are reported elsewhere [22]; this also included observation of care delivery and interviews with patients and carers, although these were not included in this formative work due to the timescales involved). Professionals were identified through snowball sampling facilitated by local study investigators. Recruitment continued until data saturation was reached. Details of participants are provided in Additional file 1. Interviews and focus groups were audio recorded, transcribed, and anonymised prior to analysis.

We used realist methodology [24, 25] to identify both causal factors and change mechanisms. This is an approach to literature review and data analysis which seeks to answer the question ‘what works for whom under what circumstances, how and why’, describing mechanisms which produce particular outcomes in specific contexts [26]. Members of the qualitative team (AW, CB) generated first “if-then” statements and grouped these according to emerging themes [27]. We refined the if-then statements, looking for data that could be interpreted as a causal factor or a change mechanism. We expressed these using the realist framework of Context, Mechanism or Outcome [25], with mechanisms further divided into ‘resource’ (the intervention component added) and ‘reasoning’ (what change this resource will produce) [28]. Finally, we presented these initial Context-Mechanism-Outcome configurations (CMOcs) to a panel of clinicians (LA, RB, CF, SP, LR) and further refined them based on their feedback. This framework formed the basis for extracting data from the literature. For examples of finalised CMOcs, see Table 1.

**Table 1:** Optimising the circumstances of rehabilitation for people with dementia: CMOcs, consensus statements and outcomes

	CMOc	Consensus statements	Outcome	Operationalisation
CMOc1	Context: cognitive impairment may limit the ability of people with dementia to articulate pain Mechanism (resource): staff use non-verbal pain signifiers and/or give blanket pain relief Mechanism (reasoning): people with dementia are not in pain Outcome: capacity to engage with an intervention increases	Tools which assess non-verbal signs of pain should be used	Agreed in round 1 (93%)	• Checklist of Nonverbal Pain Indicators (CNPI) [44, 89] included in assessment document • Pain management included in staff training
CMOc2	Context: cognitive impairment may limit the ability of people with dementia to adapt to and cope with new environments Mechanism (resource): intervention assessment and delivery takes place in appropriate, accessible and familiar environments Mechanism (reasoning): people with dementia feel comfortable and less distracted Outcome: anxiety and challenging behaviours are reduced	The intervention should primarily take place in the patient’s home	Agreed in round 1 (86%)	Intervention delivered mainly in patient’s home
CMOc3	Context: the role of comorbidities may be underestimated in dementia Mechanism (resource): holistic biopsychosocial assessment is employed Mechanism (reasoning): staff understand the range of factors contributing to falls and are able to treat comorbidities more effectively Outcome: falls risk may be reduced and recovery enhanced in patients with dementia	A continence assessment is required	Agreed in round 1 (79–100%)	All included in assessment document (see Table 2 below)
		An assessment of comorbidities is required		
		An osteoporosis risk assessment is required		
		A vision assessment is required		
		A medication review is required		
		An assessment of challenging behaviour is required		
		Formal assessments of gait and balance should be carried out by the Timed Up and Go (TUG) test [90]	No consensus after 2 rounds (54% & 62%)	
		All patients require attendance for a lying and standing blood pressure (BP)		

#### Effectiveness review

This has been reported elsewhere [5]. The review could not draw definitive conclusions, since few interventions were aimed at patients with dementia, and those that were focused mainly on hip fracture. It therefore indicated that the development of a new intervention was warranted.

#### Realist synthesis

The protocol for the realist synthesis was registered with PROSPERO (CRD42016040059).

##### Search strategy

Searches were limited to English. Initially we undertook a comprehensive search (SR). This took place in November 2015, and was designed to provide a clear understanding of the interaction between interventions, characteristics of people with dementia and contextual factors around a fall. Iterative targeted searches aimed to build on that understanding and were completed by March 2017 (FB). As the aim of the paper is to describe the intervention development process as it occurred, the searches have not been updated.

**Fig. 2:**
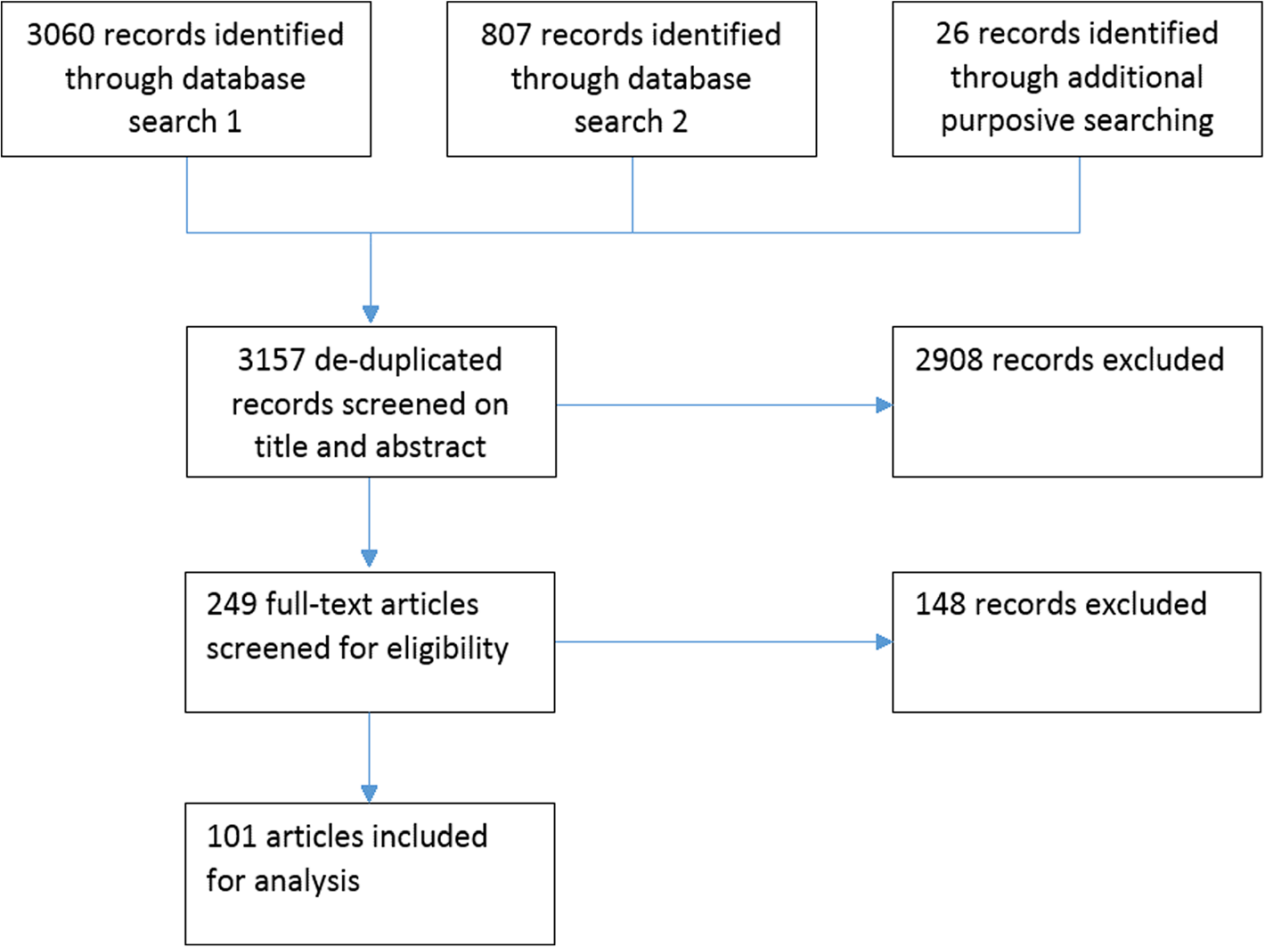
Diagram of the search, screening, selection and extraction process

Comprehensive searches were conducted in MEDLINE, CENTRAL, Health Management Information Consortium, EMBASE, CINAHL, Web of Science, Allied and Complementary Medicine Database, and Physiotherapy Evidence Database (PEDro) (see Additional file 2 for an example search strategy). Trials registers were searched, but further grey literature searching was not conducted. Results from all databases were imported to Endnote. Targeted searches took place in MEDLINE and CINAHL on EBSCO (see Additional file 3 for an example targeted search strategy). Additional papers were identified through citation chaining of included papers and relevant systematic reviews and hand searches. Figure 2 demonstrates the flow of studies.

##### Data extraction and CMOc refinement

Data were extracted from included papers using a bespoke online form. This included methodology, appraisal using the Mixed Methods Appraisal Tool [29]; an intervention description, as applicable, using the TIDieR framework [30]; and evidence describing contexts, mechanisms or outcomes. Data were extracted by two reviewers independently, one clinician (LA, BB, CF, SP, SL) and one non-clinician (CB, FB, CS, AW). Data were discussed at a meeting of reviewers and disagreements resolved. The qualitative team (CB, CS, AW) analysed and summarised the data. Following this process, the wording of each CMOc and the set of themes were refined (CB, CS, AW). The process was repeated for the additional papers identified through targeted searches and citation chaining.

### Delphi consensus process

We convened a multidisciplinary panel of 24 expert health and social care professionals (see Additional file 1) to review the initial findings and make recommendations regarding the design of the complex intervention using a modified Delphi panel approach [31–33] (see Additional file 4). Panellists were selected who (1) represented of a range of stakeholder groups identified to be important to the care of people with dementia who fall; (2) were in contact with people with dementia who fall and/or (3) had relevant academic expertise; (4) and were able to attend face-to-face meetings. The consensus panel meetings were audio recorded (with the consent of participants) and transcribed for analysis.

#### Consensus panel meeting 1

Prior to the first meeting (March 2017), the panel received summaries of the qualitative work, effectiveness review, and the realist synthesis. At the meeting, members were split into groups to discuss three key aspects of the intervention: feasibility and setting; content; and outcome measures. Each group discussed all issues. Key points from the discussions were fed back and areas of initial agreement and dissent were identified.

#### Delphi surveys

Following the first consensus panel meeting, a series of statements were identified and sent to panel members via an online survey tool. Members were asked to respond to specific questions regarding feasibility of the setting; staffing and training requirements; components of the intervention; and outcome measures for the feasibility study. A threshold of two-thirds agreement of those completing the survey was chosen to represent consensus. Responses were received from 14 panel members.

Since consensus was not achieved on all items, a second round of the survey was conducted which included the results of the first round. This gave members the opportunity to revise their responses. Responses were received from 13 panel members.

All respondents completed all items in both rounds of the survey. To facilitate free expression of opinion, only the independent moderator (BE) could access non-anonymised data. Statements included in the survey along with consensus results are given in Additional file 5.

#### Stakeholder feedback

In parallel with the surveys, additional focus groups and interviews were conducted with a range of stakeholders to explore the feasibility and acceptability of the draft intervention (see Additional file 1 for details of participants). These were invited from the pool of participants who took part in the previous qualitative work, supplemented by snowball sampling of professionals and additional patients and carers recruited via the North East and North Cumbria CRN Case Register. Interviews and focus groups were audio-recorded, transcribed, and anonymised prior to thematic analysis.

#### Consensus meeting 2

At the second meeting (June 2017), panel members considered the draft protocol for the feasibility study; results of stakeholder feedback on the proposed intervention; and the proposed roles of members of the multidisciplinary team (MDT). Small group discussions were facilitated as at the first meeting.

### Collation of results and development of a logic model

We collated the findings of the final round of the Delphi survey, consensus panel discussion, and stakeholder feedback to finalise the protocol for the feasibility study and model the intervention. The logic model was developed by the qualitative team (CB, AW) informed by existing logic model templates [34, 35] and was discussed by the Trial Oversight Committee (TOC).

### Preparation of intervention resources

Three specific resources needed to implement the intervention were identified from the protocol and logic modelling process: an assessment document, a manual and a staff training programme. These were developed by the study team (LA, CB, EF, AS, AW) with reference to the final consensus statements, protocol, and logic model and were further discussed by the TOC and all co-investigators.

## Results

Nine CMOcs were identified as key components of a new intervention for people with dementia following a fall. These were grouped into three themes:

- Ensuring that the circumstances of rehabilitation are optimised for people with dementia (CMOcs 1–3)
- Compensating for the reduced ability of people with dementia to self-manage (CMOcs 4–6)
- Equipping the workforce with the necessary skills and information to care for this patient group (CMOcs 7–9)

In presenting each CMOc, we synthesise evidence from the literature, map relevant consensus statements, describe how intervention components agreed by the consensus process were operationalised for the DIFRID intervention, and present results of stakeholder feedback. A fourth theme, covering practicalities relating to intervention delivery and evaluation, is also discussed.

Quotations presented are identified by a unique participant ID. Additionally, role and service type is provided for professionals. All identifying information, including location, has been removed to maintain confidentiality.

### Theme 1: ensuring that the circumstances of rehabilitation are optimised for people with dementia

This theme concerns the ways in which broader contextual factors, such as setting and comorbidities, can affect the engagement of people with dementia in interventions. The outcomes of the consensus survey and operationalisation of each CMOc are shown in Table 1.

#### CMOc1: managing pain

Pain is associated with impaired mobility and physical functioning [36–38] and increased agitation and aggression [39–42] in people with cognitive impairment or dementia. Sleeping and mood disorders in people with dementia have also been linked to higher pain levels [43]. People with dementia who are in pain may therefore find it more difficult to engage fully with an intervention. However, recognising pain in people with dementia can be challenging as they may be unable to verbally communicate their pain [44].

The consensus panel agreed that identifying pain should be part of the DIFRID intervention. Stakeholders highlighted the complexities of assessing pain in people with dementia:

*There are so many different implications. It is not just about us scoring pain. If you are talking about pain assessment, you need to do it properly. That, again, is multi-factorial. You need to use the appropriate pain scoring. If you are talking about people who have got moderate dementia who are cognitively impaired, you need to be thinking about something like the Abbey Pain Scale or something like that. It is not verbal. It is behavioural, body language, facial expressions, all that sort of stuff.*

***(Prof 122, pain nurse, focus group with specialist nurses)***

#### CMOc2: ensuring a supportive environment

People with dementia may become distressed in an unfamiliar environment, resulting in an exacerbation of symptoms [45]. Moreover, since people with dementia may find it difficult to articulate basic needs, such as hydration, these may go unrecognised by staff [45]. Carers in one qualitative study described negative experiences of hospitalisation, such as a deterioration in patients’ health, and were keen to avoid readmission [46]. Home-based exercise interventions have been shown to be feasible for at least some patients with cognitive impairment and hip fracture [47–50], though some studies reported problems with adherence [51]. Literature relating to patients with other fall-related injuries was not found.

The consensus panel agreed that the home environment would be the most appropriate location for the DIFRID intervention. Stakeholder feedback on this aspect of the intervention was generally positive, although some stakeholders highlighted the need for flexibility:

*I don’t know how that would fit in, because we used to enjoy walking, you see, up in the hills, and I’m not quite sure how that would fit in with physio in the home.*

*(Interview, Patient 15 and* ***Carer 15*** *)*

The intervention, therefore, can be delivered in the most appropriate environment for the activities and goals identified by participants.

#### CMOc3: adopting a holistic approach

Holistic assessments to discover and manage falls risk factors emerged as an important theme. Comorbidities that increase mortality risk during and after hospitalisation for hip fracture in older people may go unrecognised and undiagnosed [45, 52, 53]. Psychosocial factors, such as depression [53, 54] and social isolation [54], may also be important for the wellbeing and recovery of people with dementia following a fall. Holistic assessments, such as Comprehensive Geriatric Assessment (CGA), have been shown to improve outcomes for people with cognitive impairment or delirium who have fallen [53–55]. Holistic assessment may also aid patient and caregiver understanding of the causes of falls [56] and facilitate early intervention for other health issues which might otherwise undermine therapy [57].

Stakeholders suggested including a social worker in the DIFRID MDT to facilitate assessment of social circumstances:

*I think it’s really important that people get a review of their social circumstances, especially if they’ve had a fall. Sometimes […] the reason that they’ve fallen is that they’re trying to do something that they would benefit from having a care package to prevent them having to do themselves.*

***(Prof 71, reablement support worker, focus group at specialist inpatient rehabilitation unit)***

The consensus panel subsequently agreed that a social worker should be available on referral. Additional areas for assessment suggested by stakeholders included: foot assessment; nutrition; frailty; existing equipment and aids; and a detailed cognitive profile. Details of the assessment, conducted using skilled observation or verbal report from patient and carer, are shown in Table 2.

**Table 2:** Sections of the assessment and intervention document

Generic assessment (by physiotherapist or occupational therapist)	
Falls history	
Falls risk assessment (including fear of falling, nutrition, fluid intake, pain, urinary incontinence, bowel incontinence, supportive footwear, visual impairment not corrected with glasses)	
Past medical history and comorbidities	
Medication	
Current activity levels	
Challenging behaviour and sleep disturbance	
Assessment of the needs of the informal carer	
Current mobility	
Physiotherapy assessment	Occupational therapy assessment
Posture and general observations of pain, sensation and tone	Details of home environment
Lying and standing BP	Self-care and productivity
Range of movement	Cognition
Muscle power	Task observations
TUG	Functional difficulties relating to spatial awareness, vision and hearing
Intervention planning	
Needs list	
Action planning and patient goals	
MDT record	
Referrals	

Stakeholders emphasised the need to interpret the results of holistic assessment and identify clear processes for addressing issues raised:

*For example, incontinence, you know, you are not going to engage someone in an exercise programme, or encourage them to stabilise their gait, their balance or posture if actually their real problem is they are retaining urine. They are getting overflow, and when they stand up to go they have a real sense of urgency and they are desperate. You can put in every intervention you like. Unless you address that problem… You need someone who is going to think about that, and understand what is going on. The reason they are in a hurry to get up and go to the loo is not because they are going frequently. They frequency is due to another problem that hasn’t been picked up.*

***(Prof 122, pain nurse, focus group with specialist nurses)***

In developing the assessment and intervention materials, we therefore added a section dedicated to referrals for issues identified during assessment, and tasked the MDT with reviewing this. A substantial component of the DIFRID staff training programme focused on using the assessment document and managing any problems identified.

### Theme 2: compensating for the reduced ability of people with dementia to self-manage

This theme concerns the ways in intervention delivery can be adapted to compensate for the symptoms and challenges of dementia. The outcomes of the consensus survey and operationalisation of each CMOc are shown in Table 3.

**Table 3:** Compensating for the reduced ability of people with dementia to self-manage: CMOcs, consensus statements and outcomes

	CMOc	Consensus statements	Outcome	Operationalisation
CMOc4	Context: cognitive impairment may limit the ability of people with dementia to comply with instructions and form habits Mechanism (resource): staff tailor the intervention (e.g. exercises) to the circumstances of people with dementia and embed it in their existing routines Mechanism (reasoning): intervention becomes routine and habitual Outcome: more successful rehabilitation can be achieved	Interventions should be based on goals set by the patient and carer	Agreed in round 1 (86–100%)	• Goal Attainment Scaling [91] (GAS) implemented • Compass of Life [92] included to assist in goal identification
		Therapists should work with service users to minimise the risk of falling, as this may improve confidence and enable realistic risk taking.		Falls risk assessment included
		Therapists should help the service user and caregiver to develop a meaningful programme of activities		• Assessment records personal preferences, routines, and priorities • Therapists develop programme of meaningful activities based on information gathered
		Therapists should undertake observed activities with the service user to facilitate new learning		Included in assessment
		Exercise interventions should be informed by evidence based formats such as the Otago programme but tailored to the circumstances of people with dementia and embedded in their daily life	Agreed in round 2 (69%)	• During training, staff are encouraged to use evidence-based formats creatively • Training also includes advice on creating programmes and embedding them into routines • Coloured paper provided for embedding strategies
CMOc5	Context: cognitive impairment may limit the ability of people with dementia to self-manage changes in circumstances Mechanism (resource): ongoing follow-up is provided Mechanism (reasoning): staff are able to reinforce previous interventions and adapt them to meet changing needs Outcome: improvements in mobility are sustained and new falls risks reduced	The total number of physiotherapy sessions available in the first 3 months (including sessions delivered by a support worker) should be 16, 20 or 24	No consensus after 2 rounds (31–62%)	Implemented 2 assessment sessions and maximum 22 therapy sessions delivered by a mix of OT, physiotherapist and support worker
		The total number of occupational therapy (OT) sessions available in the first 3 months should be 3–4		
CMOc6	Context: the burden on informal carers is high when caring for relatives or friends with dementia who are at risk of falling Mechanism (resource): carer support and education is provided Mechanism (reasoning): carer stress is reduced and skills increased Outcome: carers’ capacity to assist with the delivery of interventions increases	Carer stress should be routinely assessed	Agreed in round 1 (93–100%)	• Carer stress included in assessment • Training emphasises ensuring carers have capacity to be involved
		Therapists should facilitate caregivers, family and friends to adopt a positive approach to risk		• Training includes advice on carer education, including accepting ‘positive risk’ • Carer education leaflets provided for dissemination [93, 94]
		Intervention staff should be able to provide basic carer education & support, referring to other agencies as needed	Agreed in round 2 (77%)	

#### CMOc4: embedding interventions in day to day life

Individually tailoring exercises to the preferences, interests, and physical and cognitive abilities of people with dementia has been described as ‘vital’ to successful interventions for this patient group [49]. Cognitive impairment may affect the ability of patients to follow instructions and consequently, rehabilitation success [47, 51, 53–55, 58–62]. However, some people with dementia may have relatively well preserved procedural memory which may enable them to form new habits [54]. ‘Embedding’ interventions into existing routines could also help make them habitual [63–65]. Effective tailoring requires specialised training for staff and carers involved in intervention delivery [49, 66]; including a staff training component in the intervention was therefore seen as essential (see CMOc8).

Stakeholders agreed with the consensus panel’s recommendation to use the principles underlying the Otago exercise programme (i.e. individually tailored; progressive; stable and sustainable; and using walking alongside strength and balance) [12], although they indicated that implementation of this programme is often inconsistent with the recommended format due to resource limitations. Stakeholders emphasised that meaningful activities should include mental and social stimulation as well as physical activity:

*This gentleman had really bad dementia. He had sundown so he was up all night. The family came in, and we had a game of dominos. I couldn’t communicate with him. You bring out the dominos and he won every time. It was like a different person came out in that dominos. […] Then, by making him stay awake all day and doing meaningful activities to keep him active, he was more likely to sleep at night. […] He is not getting up and falling over.*

***(Prof 121, focus group with specialist nurses)***

Music and dance were also considered particularly valuable. Exploring the barriers (including cultural barriers) to meaningful activity was identified as one way to increase the likelihood of successfully engaging patients in new activities.

While setting patient-centred goals achieved a high level of consensus among the panel, some stakeholders had reservations about how this might work in practice:

*I don’t think I could do it. Like, make a cup of tea. I wouldn’t trust myself.*

***(Interview, Patient 13)***

Professional stakeholders also identified potential problematic elements of goal setting, including the difficulty of engaging people with dementia in setting goals, the potential for them to lose interest in things they previously enjoyed, and ensuring goals were those of the patient and not only the carer.

#### CMOc5*:* providing ongoing support

One quarter (24%) of re-admissions following hip fracture surgery are due to ‘failure of rehabilitation’—including deterioration, further falls, and inability to cope [57]. This suggests that the duration and/or dosage of existing rehabilitation may be insufficient. As people with dementia typically have difficulties with problem solving and self-management, providing only short-term interventions may be particularly problematic for this patient group. Professionals in the initial qualitative study felt that existing interventions were often too short and lacked continuity in content and staffing [21]. They proposed regular follow-up and review to help identify new problems or relapses and maintain continuity of care.

The intensity and duration of the intervention proved to be the most contentious aspects of the intervention among the consensus panel and stakeholders. Ultimately, the consensus panel were constrained by the realities of the project timescale, which could only accommodate a 12-week intervention period. Providing on-going support was therefore not feasible. However, the panel allowed for up to a total of 22 intervention sessions over 12 weeks; this is substantially more than is provided by many existing services, which our initial qualitative work found were typically provided for between 2 and 6 weeks. The Delphi survey therefore included questions on setting appropriate boundaries.

All groups of stakeholders stressed the need to tailor the number of intervention sessions to the individual. However, community-based professionals, particularly those in rural areas, raised concerns over the feasibility of delivering this number of sessions both within and outside the context of a trial. The duration of individual sessions and the intervention overall were also queried by some participants:

*You need at least, you know, half of that time even strike up a rapport, for them to remember, possibly, who you are, for you to engage with the carer, and that’s before you’ve even done anything and before you’ve even assessed the person or given them any intervention. That’s every time, because every time is like a new time.*

***(Prof 124, physiotherapist, focus group with community health and social care professionals)***

Other participants questioned whether the allotted 12 weeks would be long enough for all referrals to have been acted upon and for alternative services to have been put in place to provide ongoing support. Carers also expressed concern about what would happen after the intervention:

*That would be my only concern. You're leaving people, then, in limbo. You're offering them something that isn't there anymore. It was there, but ‘oh, that's not there now’.*

***(Interview, Carer 12)***

The intervention therefore includes mid-point and final review sessions, where intervention staff check the status of referrals, treat new issues arising during the intervention period, and signpost participants to other relevant services (such as activity groups) to help maintain progress after the completion of the 12-week intervention.

#### CMOc6: involving carers in intervention delivery

The involvement of family carers is frequently recommended to improve adherence and outcomes of interventions [53, 54, 56, 67]. However, this implicitly assumes that carers have capacity and the skills to assist in intervention delivery. Many family carers report feeling isolated, helpless, and overstretched by providing care as well as dealing with their own health problems and other commitments [46, 56]. Having realistic expectations of carers is therefore important [68]. Factors shown to facilitate carer involvement include exploring concerns about time requirements and disruption to routines [69], understanding that carers may have difficulty of acknowledging that they need help [69], and explicitly discussing potential benefits of a rehabilitation intervention to both people with dementia and carers [69–72]. Carers may also benefit from interventions tailored to their own needs [66, 73–76]. Carer behaviours, such as preventing the person with dementia from moving around in order to avoid falls, can negatively influence the relationship between carer and patient [46] and impede recovery.

The consensus panel agreed that educating patients and carers about positive risk and falls prevention was important. This was also deemed beneficial by stakeholders:

*The physios and OTs […] can assess whether or not that person needs signposting to have some more help. I'm not saying you'd have to have somebody come in with them and do the carer support, but I do think that training them what to look out for, carer fatigue and the stress side of things.*

***(Interview, Carer 12)***

However, professionals also emphasised the importance of ensuring that carer needs do not overshadow those of the patient. To address this concern, the DIFRID training programme includes advice on managing triadic consultations.

### Theme 3: equipping the workforce with the necessary skills and information to care for people with dementia

This theme concerns both the training needs of staff and the practical organisation of interventions to improve information gathering and communication. The outcomes of the consensus survey and operationalisation of each CMOc are shown in Table 4.

**Table 4:** Equipping the workforce with the necessary skills and information to care for people with dementia: CMOcs, consensus statements and outcomes

	CMOc	Consensus statements	Outcome	Operationalisation
CMOc7	Context: cognitive impairment may limit the ability of people with dementia to pass on information Mechanism (resource): staff use multiple sources of information including carers and direct observation Mechanism (reasoning): staff gain a better understanding of the individual Outcome: staff are able to provide appropriate, tailored care	Assessment should involve multiple sources of information including information from carers	Agreed round 1 (93–100%)	The assessment (Table 2) includes all of these components
		Assessment should include direct observation		
		A home hazard assessment should include a walk around the house to determine where actual falls have occurred and negotiate how these might be reduced		
CMOc8	Context: current staff knowledge of, and attitudes to, dementia are variable Mechanism (resource): increased dementia training is provided Mechanism (reasoning): staff gain skills in and understanding of rehabilitation for people with dementia Outcome: staff ability and willingness toengage with people with dementia is enhanced	Tier 2 training is required for intervention staff	Agreed round 2 (85%)	This was deemed unfeasible in the time available. A tailored training programme was developed, including items from tier 2 training.
		Training needs to include how to tailor an intervention for people with dementia.	Agreed round 1 (100%)	Training includes this
		Training needs to include advice on how to engage and motivate people with dementia.		Training includes this
		Training should include on the job role modelling		This was deemed unfeasible in the time available. Training delivered by therapists with experience in working with people with dementia, who were available remotely for advice.
CMOc9	Context: care pathways are often unclear Mechanism (resource): a centralised, collaborative pathway is developed and disseminated Mechanism (reasoning): staff are better equipped to refer to the most appropriate services Outcome: service users receive better treatment	The setting of the intervention should make use of existing pathways only when referral from the team deems it would be useful for the individual	Agreed round 1 (85.7–100%)	Assessment document includes tracking referrals that are decided by MDT
		A multidisciplinary team (MDT) meeting should be available if needed		• MDT composition agreed as physiotherapist, OT, support workers and geriatrician, with a general nurse available where the team already included this. Community psychiatric nurse (CPN), social workers, reablement workers, old age psychiatrists and podiatrists accessible by referral. • MDT meetings available at beginning and middle of intervention period.
		Therapists should offer service users information on assistive devices and facilitate delivery		This is flagged in the assessment document and available when needed

#### CMOc7: developing a detailed understanding of the patient

A detailed understanding of the patient is fundamental providing tailored, person-centred care in dementia. As people with dementia may struggle with giving full and accurate medical histories [45, 57, 77], direct observation of the patient in the environment in which they fell was recommended by professionals in the initial qualitative study [22]. Additional context or confirmation can be provided by carers [45, 46] or patients’ GPs [77]. Drawing on carer expertise to facilitate the care of people with dementia in hospital has been shown to be effective in reducing agitation and distress and improving carer satisfaction, though levels of patient satisfaction were not reported [60].

Stakeholder feedback about this aspect of the intervention was positive, particularly around using carers as information sources. Professionals also agreed that assessment by observation was important, particularly with regard to how participants get around the house.

#### CMOc8: equipping staff members with appropriate skills

Staff members may lack specific training in working with people with dementia and their families, and negative views about people with dementia and their ability to participate in an intervention have been reported [66, 74–76]. Several authors recognised the value of providing specialised training of staff to work with older adults and people with dementia, though few provided detailed information on the content of such training [77–79]. Data from the qualitative study suggests training should cover dementia-specific adaptation to practice, as well as challenging negative attitudes towards those with dementia [22]. Training in how best to engage with carers could also be beneficial [60].

Stakeholders identified training as one of the most crucial components of the intervention:

*What’s jumping out to me is the dependence on the staff training. From a list of interventions none of those are really, hugely, a step away from what we cover. But I know, definitely, still in our organisation staff still need to understand that you can’t deliver the same package to someone with a physical condition as to somebody with some challenges, whatever they are.*

***(Prof 35, dementia and falls co-ordinator, focus group with community health and social care professionals)***

#### CMOc9: improving pathways and referral

Collaboration between professionals is an important factor in whether patients receive effective treatment [52, 80, 81]. A range of social and contextual factors influences decisions to refer to services, including lack of confidence in the service provided, reluctance to share responsibility for patient care, or a perception that the patient would not benefit from the service [57, 79]. The initial qualitative study found staff often lacked knowledge of local services for people with dementia with fall-related injuries [22]; however, this evidence suggests that a simple lack of knowledge may not be the only barrier to successful care. The advantages of formalised care pathways include increasing efficiency of diagnosis and beginning treatment, increasing consistency of care, reducing risk of errors, reducing costs, and improvements in staff knowledge and team relations [82, 83]. Developing an evidence-based pathway requires collaboration and input from stakeholders including health professionals, patients and family members [84]. Ultimately, the consensus panel agreed that developing a new care pathway for fall-related injuries in dementia was outside of the scope of this study, though e issues of communication and referral were addressed, and the proposed MDT meetings were seen as a way of maximising use of existing pathways.

Stakeholders raised concerns over the feasibility of organising MDT meetings, particularly in rural areas. While the use of technology could potentially enable virtual MDT meetings, issues were raised over security and the need for encryption. Overall, professional stakeholders identified a need to clarify the roles of each member of the DIFRID MDT; this was subsequently discussed at the second consensus panel meeting. Stakeholders additionally suggested including dietitian/nutritionist; Alzheimer’s Society outreach workers; and advocacy advisers in the MDT. Potential benefits of including a community psychiatric nurse (CPN) in the MDT were: (i) access to mental health records, which provided information about dementia, medications and other interventions; (ii) the potential for rapid referrals and specialist support; and (iii) the potential role of CPNs in reviewing medications:

*[The CPN at our service] can pull up information on where people are at in terms of the support and input that they have had already when they’ve last been reviewed at memory services. She can review their medications as well which can be really helpful.*

***(Prof 71, reablement support worker, focus group at specialist inpatient rehabilitation unit)***

These additional staff have not been included in the MDT, but intervention materials signpost therapists to refer to them as required.

### Theme 4: intervention delivery and evaluation

The remaining consensus statements concerned issues of practicality and feasibility for the pilot study (for example, inclusion criteria, recruitment, and outcome measures).

#### Design and feasibility

In round one of the Delphi survey, 93% of the consensus panel agreed that a complex intervention was needed. It was deemed feasible to recruit 10 patients from each of three sites to the feasibility study. Defining the inclusion criteria for the intervention proved more contentious among the panel. The original brief for this study was to design a new intervention for people with dementia following a fall-related injury. In the initial interviews and focus groups professionals argued that early intervention, prior to significant injury, would be more beneficial. The consensus panel also agreed that the intervention should include patients with non-injurious falls. However, the TOC subsequently strongly recommended amending this to a fall for which healthcare attention was sought. Consensus regarding the time period within which patients had to be recruited following a fall was not reached after two rounds of surveys. Following discussion at the second meeting, it was agreed that patients could be recruited up to 1 month after the index fall.

#### Outcome measures

One aim of the feasibility study is to assess the suitability and acceptability of outcome measures. While the number of falls was seen as the most appropriate outcome measure by the consensus panel, other stakeholders expressed reservations about the sensitivity of this measure:

*There are maybe subtleties there, from my thinking, that if it was just based on that what might seem like a fail is actually an improvement because the person does feel more confident, is doing more things but is having non-injurious falls as a side-line.*

***(Prof 35, dementia and falls co-ordinator, focus group, community health and social care professionals)***

To address these concerns, a range of outcome measures are being used in the feasibility study, including measures of function, quality of life and carer burden [23].

#### Logic model

**Fig. 3:**
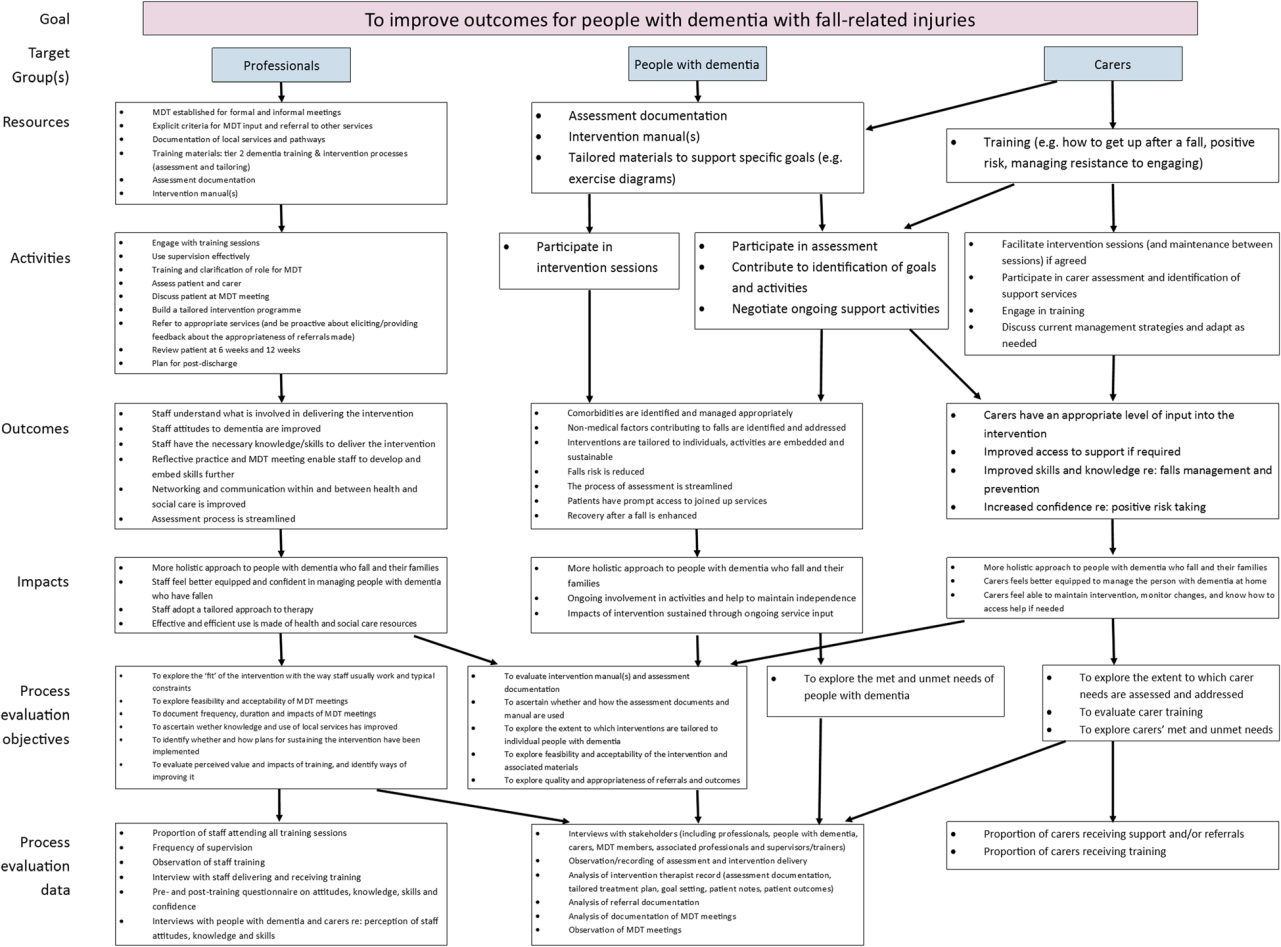
Logic model

A logic model (Fig. 3) demonstrates the flow of intervention activities to meet project goals.

## Discussion

We used a mixed-methods approach to develop the DIFRID intervention. We identified causal factors and change mechanisms through analysis of qualitative data collected in an earlier phase of the study and a realist synthesis of the literature. This is summarised in three broad themes:

- Ensuring that the circumstances of rehabilitation are optimised for people with dementia
- Compensating for the reduced ability of people with dementia to self-manage
- Equipping the workforce with the necessary skills and information to care for this patient group.

An expert panel considered how best to translate these concepts into a new intervention. Consensus among the panel on which components should be included was achieved through two rounds of a Delphi survey. This process allowed us to integrate practical, empirical data from experts and practitioners with evidence from previous studies to create a robust, theoretically-informed design for a new intervention.

Despite the structured approach to intervention development, not all of the CMOcs that emerged from the initial synthesis were equally present in the consensus surveys. CMOc5, for example, which concerned ongoing support and follow-up of people with dementia, was deemed beyond the scope of this study; panel members expressed concerns regarding practicality and feasibility of engaging in such follow-up when working within constraints such as funding, existing multidisciplinary teams, existing service provision, and the 12-week limit of the trial. Moreover, for practical reasons relating to costing the intervention, it was difficult to allow the number of sessions to be open-ended. The 12-week intervention period is quite short in comparison with some trials of exercise in older people [85]. However, there are a number of trials which have successfully used this intervention period. In our development work, we found people with dementia received few interventions, often limited to 2 or 6 weeks, so a 12-week intervention is a substantial improvement [21]. Additionally, at the end of the DIFRID intervention therapists are encouraged to refer participants on to community falls groups or other appropriate ongoing services. It is possible that in future development of this intervention we could consider extending the intervention beyond 12 weeks but this will not be possible within the funding for our planned feasibility trial.

CMOc9 refers to the creation of a centralised pathway, which was similarly considered beyond the scope of the study; instead, the consensus process focused on improving communication within and between staff. Not all components were systematically translated and included in the Delphi survey; this led to the omission of a statement relating to blanket pain relief as described in CMOc1, for example. Potential pitfalls associated with this kind of iterative process of intervention development therefore include ensuring follow-through of ideas at each stage and the potential disconnect between theoretical ideals and what is considered practical and feasible in everyday practice. Though we aimed to follow processes for intervention development [19, 20], these were not always smoothly navigated from one stage to the next. A more rigorous approach to the process of operationalising CMOcs to Delphi survey to final intervention could help to mitigate some of these pitfalls.

The final intervention agreed is a home-based, tailored therapy intervention delivered by an MDT that includes physiotherapists, occupational therapists, therapy assistants, and a geriatrician (see Additional file 6). Up to two assessment sessions and 22 intervention sessions will be available. The resources developed include an intervention manual for staff; a holistic assessment document to help staff to tailor the intervention; and a staff training programme [23]. This is in concordance with guidelines that recommend multifactorial interventions for falls in older people [86]. Though some evidence suggests that such interventions are not effective in people with dementia [9–11], it is hoped that the individually tailored, embedded approach will help to mitigate some of the factors affecting intervention success among this patient group [49].

The intervention that has been developed is novel in that it is tailored to the needs of people with dementia and addresses both rehabilitation and the prevention of future falls in people with dementia. While we are aware of a current study examining enhanced recovery of confused patients following hip fracture [6], this focuses on a single type of injury. Other current studies are focusing on falls interventions for people with dementia, but are not targeted at those who have already had an injurious fall [87, 88]. The DIFRID intervention therefore targets a neglected group, and could potentially clarify whether the preventive component is effective in patients who have already fallen.

### Strengths and limitations

A strength of this project lies in the theoretically and empirically-informed intervention development process. While a response rate of 58% was achieved for the consensus surveys, not all panel members attended the consensus meetings. Furthermore, the panel did not include patient or lay representatives. The Delphi approach seemed less accessible for social care professionals, as evidenced by difficulty recruiting panel members and engaging them in the surveys. These factors may have implications for the results. However, the iterative nature of our approach to identifying causal factors and change mechanisms and stakeholder feedback process means that the opinion of these stakeholders has been considered in other aspects of the development process. While the effectiveness review highlighted the scarcity of evidence and underpinned the need to develop a new intervention, it was of limited value in the process of intervention development. In contrast, the broader, pragmatic realist approach helped to consider underlying mechanisms, and inform intervention content and delivery.

## Conclusions

A new intervention has been developed to help people with dementia following a fall requiring healthcare attention. We are currently assessing the feasibility and acceptability of the DIFRID intervention from the perspectives of all stakeholders. If appropriate, the findings will be used to refine the intervention, and then explore whether it merits rigorous evaluation [19].

## Additional files

Additional file 1: Professional participants. Table showing numbers of participants from various health and social care professions in each stage of the project. (DOCX 12 kb)

Additional file 2: Comprehensive search strategy. Medline literature search strategy for the phase 1 comprehensive search. (DOCX 13 kb)

Additional file 3: Targeted search strategy. Example Medline literature search strategy for the phase 2 targeted searches. (DOCX 12 kb)

Additional file 4: Delphi survey. Expanded description of rationale and methodology for the Delphi survey. (DOCX 13 kb)

Additional file 5: Consensus statements. Full list of statements provided to the consensus panel along with their outcomes. (DOCX 16 kb)

Additional file 6: Final DIFRID intervention. A description of the final intervention using the TIDIeR framework. (DOCX 16 kb)

## Abbreviations

BP: Blood pressure
CGA: Comprehensive Geriatric Assessment
CMOc: Context-Mechanism-Outcome configuration
CNPI: Checklist of nonverbal pain indicators
CPN: Community psychiatric nurse
GAS: Goal attainment scaling
MDT: Multidisciplinary team
OT: Occupational Therapy/Therapist
PEDro: Physiotherapy Evidence Database
TOC: Trial Oversight Committee
TUG: Timed Up-and-Go test

## References

[1] Matthews FE, Arthur A, Barnes LE, Bond J, Jagger C, Robinson L, Brayne C, Medical Research Council Cognitive F, Ageing C (2013). A two-decade comparison of prevalence of dementia in individuals aged 65 years and older from three geographical areas of England: results of the cognitive function and ageing study I and II. Lancet.

[2] Allan LM, Ballard CG, Rowan EN, Kenny RA (2009). Incidence and prediction of falls in dementia: a prospective study in older people. PLoS One [Electron Resour].

[3] Shaw FE (2002). Falls in cognitive impairment and dementia. Clin Geriatr Med.

[4] Delbaere K, Close J, Brodaty H, Sachdev P, Lord S (2010). Determinants of disparities between perceived and physiological risk of falling among elderly people: cohort study. BMJ.

[5] Robalino S, Nyakang’o SB, Beyer FR, Fox C, Allan LM (2018). Effectiveness of interventions aimed at improving physical and psychological outcomes of fall-related injuries in people with dementia: a narrative systematic review. Syst Rev.

[6] Hammond SP, Cross JL, Shepstone L, Backhouse T, Henderson C, Poland F, Sims E, MacLullich A, Penhale B, Howard R (2017). PERFECTED enhanced recovery (PERFECT-ER) care versus standard acute care for patients admitted to acute settings with hip fracture identified as experiencing confusion: study protocol for a feasibility cluster randomized controlled trial. Trials.

[7] National Institute for Health and Care Excellence (2013). Falls: assessment and prevention of falls in older people. NICE clinical guideline 161. NICE clinical guideline.

[8] Gillespie LD, Robertson MC, Gillespie WJ, Sherrington C, Gates S, Clemson LM, Lamb SE (2012). Interventions for preventing falls in older people living in the community. Cochrane Database Syst Rev.

[9] Hedman AM, Grafstrom M (2001). Conditions for rehabilitation of older patients with dementia and hip fracture--the perspective of their next of kin. Scand J Caring Sci.

[10] Jurgens FJ, Clissett P, Gladman JR, Harwood RH (2012). Why are family carers of people with dementia dissatisfied with general hospital care? A qualitative study. BMC Geriatrics.

[11] Vaapio SS, Salminen MJ, Ojanlatva A, Kivela SL (2009). Quality of life as an outcome of fall prevention interventions among the aged: a systematic review. Eur J Pub Health.

[12] Gardner MM, Buchner DM, Robertson MC, Campbell AJ (2001). Practical implementation of an exercise-based falls prevention programme. Age Ageing.

[13] El-Khoury F, Cassou B, Charles MA, Dargent-Molina P (2013). The effect of fall prevention exercise programmes on fall induced injuries in community dwelling older adults: systematic review and meta-analysis of randomised controlled trials. BMJ.

[14] Martins AC, Santos C, Silva C, Baltazar D, Moreira J, Tavares N (2018). Does modified Otago exercise program improves balance in older people? A systematic review. Prev Med Rep.

[15] Hauer K, Schwenk M, Zieschang T, Essig M, Becker C, Oster P (2012). Physical training improves motor performance in people with dementia: a randomized controlled trial. J Am Geriatr Soc.

[16] Zieschang T, Schwenk M, Becker C, Uhlmann L, Oster P, Hauer K (2017). Falls and physical activity in persons with mild to moderate dementia participating in an intensive motor training: randomized controlled trial. Alzheimer Dis Assoc Disord.

[17] Dementia - assessment, management and support for people living with dementia and their carers [https://www.nice.org.uk/guidance/ng97]. Accessed 20 June 2018 30011160

[18] Hoddinott P (2015). A new era for intervention development studies. Pilot Feasibility Stud.

[19] Wight D, Wimbush E, Jepson R, Doi L (2016). Six steps in quality intervention development (6SQuID). J Epidemiol Community Health.

[20] Craig P, Dieppe P, Macintyre S, Michie S, Nazareth I, Petticrew M, Medical Research Council Guidance (2008). Developing and evaluating complex interventions: the new medical research council guidance. BMJ.

[21] Wheatley A, Bamford C, Shaw C, Boyles M, Fox C, Allan L. Service organization for people with dementia after an injurious fall: challenges and opportunities. Age Ageing. In press. 10.1093/ageing/afz010 PMC6503936 30921459

[22] Bamford C, Wheatley A, Shaw C, Allan LM. Equipping staff with the skills to maximise recovery of people with dementia after an injurious fall. Aging Ment Health. 2018:1–9. 10.1080/13607863.2018.1501664. 10.1080/13607863.2018.1501664 30428699

[23] Allan LM, Wheatley A, Flynn E, Smith A, Fox C, Howel D, Barber R, Homer TM, Robinson L, Parry SW (2018). Is it feasible to deliver a complex intervention to improve the outcome of falls in people with dementia? A protocol for the DIFRID feasibility study. Pilot Feasibility Stud.

[24] Wong G, Westhorp G, Manzano A, Greenhalgh J, Jagosh J, Greenhalgh T (2016). RAMESES II reporting standards for realist evaluations. BMC Med.

[25] Pawson R, Greenhalgh T, Harvey G, Walshe K (2005). Realist review - a new method of systematic review designed for complex policy interventions. J Health Serv Res Policy.

[26] Pawson R, Tilley N (1997). Realistic evaluation.

[27] Rycroft-Malone J, McCormack B, Hutchinson AM, DeCorby K, Bucknall TK, Kent B, Schultz A, Snelgrove-Clarke E, Stetler CB, Titler M (2012). Realist synthesis: illustrating the method for implementation research. Implement Sci.

[28] Dalkin SM, Greenhalgh J, Jones D, Cunningham B, Lhussier M (2015). What’s in a mechanism? Development of a key concept in realist evaluation. Implement Sci.

[29] Pluye P, Robert E, Cargo M, Bartlett G, O’Cathain A, Griffiths F, Boardman F, Gagnon MP, Rousseau MC (2011). Proposal: a mixed methods appraisal tool for systematic mixed studies reviews.

[30] Hoffmann TC, Glasziou PP, Boutron I, Milne R, Perera R, Moher D, Altman DG, Barbour V, Macdonald H, Johnston M (2014). Better reporting of interventions: template for intervention description and replication (TIDieR) checklist and guide. BMJ.

[31] Dalkey N (1969). The Delphi method: an experimental study of group opinion.

[32] Dalkey N, Helmer O (1963). An experimental application of the DELPHI method to the use of experts. Manag Sci.

[33] McMillan SS, King M, Tully MP (2016). How to use the nominal group and Delphi techniques. Int J Clin Pharm.

[34] Kellogg Foundation (2004). Logic model development guide battle creek.

[35] Dwyer JJ, Makin S (1997). Using a program logic model that focuses on performance measurement to develop a program. Can J Public Health.

[36] Schepker CA, Leveille SG, Pedersen MM, Ward RE, Kurlinski LA, Grande L, Kiely DK, Bean JF (2016). Effect of pain and mild cognitive impairment on mobility. J Am Geriatr Soc.

[37] Kolanowski A, Mogle J, Fick DM, Hill N, Mulhall P, Nadler J, Colancecco E, Behrens L (2015). Pain, delirium, and physical function in skilled nursing home patients with dementia. J Am Med Dir Assoc.

[38] Kress HG, Ahlbeck K, Aldington D, Alon E, Coaccioli S, Coluzzi F, Huygen F, Jaksch W, Kalso E, Kocot-Kepska M (2014). Managing chronic pain in elderly patients requires a CHANGE of approach. Curr Med Res Opin.

[39] Flo E, Gulla C, Husebo BS (2014). Effective pain management in patients with dementia: benefits beyond pain?. Drugs Aging.

[40] Husebo BS, Ballard C, Sandvik R, Nilsen OB, Aarsland D. Efficacy of treating pain to reduce behavioural disturbances in residents of nursing homes with dementia: cluster randomised clinical trial. BMJ (Clinical Research Ed). 2011;343:–d4065. 10.1136/bmj.d4065 PMC3137923 21765198

[41] Ahn H, Horgas A (2013). The relationship between pain and disruptive behaviors in nursing home residents with dementia. BMC Geriatr.

[42] Ahn H, Horgas A (2014). Does pain mediate or moderate the effect of cognitive impairment on aggression in nursing home residents with dementia?. Asian Nurs Res.

[43] Husebo BS, Ballard C, Fritze F, Sandvik RK, Aarsland D (2014). Efficacy of pain treatment on mood syndrome in patients with dementia: a randomized clinical trial. Int J Geriatr Psychiatry.

[44] Herr K, Bjoro K, Decker S (2006). Tools for assessment of pain in nonverbal older adults with dementia: a state-of-the-science review. J Pain Symptom Manag.

[45] Valeriani L. Management of demented patients in emergency department. Int J Alzheimers Dis. 2011; (no pagination). 10.4061/2011/840312 PMC3089912 21559185

[46] McIntyre A, Reynolds F (2012). There’s no apprenticeship for Alzheimer’s: the caring relationship when an older person experiencing dementia falls. Ageing Soc.

[47] Giusti A, Barone A, Pioli G (2007). Rehabilitation after hip fracture in patients with dementia. J Am Geriatr Soc.

[48] Wesson J, Clemson L, Brodaty H, Lord S, Taylor M, Gitlin L, Close J (2013). A feasibility study and pilot randomised trial of a tailored prevention program to reduce falls in older people with mild dementia. BMC Geriatr.

[49] Taylor ME, Lord SR, Brodaty H, Kurrle SE, Hamilton S, Ramsay E, Webster L, Payne NL, Close JC (2017). A home-based, carer-enhanced exercise program improves balance and falls efficacy in community-dwelling older people with dementia. Int Psychogeriatr.

[50] Seitz DP, Gill SS, Austin PC, Bell CM, Anderson GM, Gruneir A, Rochon PA (2016). Rehabilitation of older adults with dementia after hip fracture. J Am Geriatr Soc.

[51] Shaw FE, Bond J, Richardson DA, Dawson P, Steen IN, McKeith IG, Kenny RA (2003). Multifactorial intervention after a fall in older people with cognitive impairment and dementia presenting to the accident and emergency department: randomised controlled trial. BMJ (Clinical research ed).

[52] Tarazona-Santabalbina FJ, Domenech-Pascual JR, Belenguer-Varea AA, Rovira Daudi E (2014). The approach to patients with cognitive impairment and hip fracture: the role of orthogeriatric care. Rev Clin Gerontol.

[53] Aharony L, Sela-Katz P (2011). Depression, falls and cognitive changes among community-dwelling elderly people. Alzheimers Dement.

[54] Goldstein FC, Strasser DC, Woodard JL, Roberts VJ (1997). Functional outcome of cognitively impaired hip fracture patients on a geriatric rehabilitation unit. J Am Geriatr Soc.

[55] Marcantonio ER, Flacker JM, Wright RJ, Resnick NM (2001). Reducing delirium after hip fracture: a randomized trial. J Am Geriatr Soc.

[56] Faes MC, Reelick MF, Banningh L, de Gier M, Esselink RA, Rikkert MGO (2010). Qualitative study on the impact of falling in frail older persons and family caregivers: foundations for an intervention to prevent falls. Aging Ment Health.

[57] Nilsson I, Rogmark C (2011). Hemiarthroplasty for displaced femoral neck fracture: good clinical outcome but uneven distribution of occupational therapy. Disabil Rehabil.

[58] Deschodt M, Braes T, Broos P, Sermon A, Boonen S, Flamaing J, Milisen K (2011). Effect of an inpatient geriatric consultation team on functional outcome, mortality, institutionalization, and readmission rate in older adults with hip fracture: a controlled trial. J Am Geriatr Soc.

[59] Gonski PN, Moon I (2012). Outcomes of a behavioral unit in an acute aged care service. Arch Gerontol Geriatr.

[60] Luxford K, Axam A, Hasnip F, Dobrohotoff J, Strudwick M, Reeve R, Hou C, Viney R (2015). Improving clinician-carer communication for safer hospital care: a study of the ‘TOP 5’ strategy in patients with dementia. Int J Qual Health Care.

[61] Raivio M, Korkala O, Pitkala K, Tilvis R (2004). Rehabilitation outcome in hip-fracture: impact of weight-bearing restriction - a preliminary investigation. Phys Occup Ther Geriatr.

[62] Toulotte C, Fabre C, Dangremont B, Lensel G, Thevenon A (2003). Effects of physical training on the physical capacity of frail, demented patients with a history of falling: a randomised controlled trial. Age Ageing.

[63] Judah G, Gardner B, Aunger R (2012). Forming a flossing habit: an exploratory study of the psychological determinants of habit formation. Br J Health Psychol.

[64] Fleig L, McAllister MM, Chen P, Iverson J, Milne K, McKay HA, Clemson L, Ashe MC (2016). Health behaviour change theory meets falls prevention: feasibility of a habit-based balance and strength exercise intervention for older adults. Psychol Sport Exerc.

[65] Lally P, van Jaarsveld Cornelia HM, Potts Henry WW, Wardle J (2009). How are habits formed: modelling habit formation in the real world. Eur J Soc Psychol.

[66] de Rotrou J, Cantegreil I, Faucounau V, Wenisch E, Chausson C, Jegou D, Grabar S, Rigaud AS (2011). Do patients diagnosed with Alzheimer’s disease benefit from a psycho-educational programme for family caregivers? A randomised controlled study. Int J Geriatr Psychiatry.

[67] Ritter MA, Harty LD (2004). Total joint replacement in patients with dementia syndromes: a report of thirteen cases. Orthopedics.

[68] Isbel ST, Jamieson MI. Views from health professionals on accessing rehabilitation for people with dementia following a hip fracture. Dementia (London). 2016. 10.1177/1471301216631141 26843421

[69] Murphy MR, Escamilla MI, Blackwell PH, Lucke KT, Miner-Williams D, Shaw V, Lewis SL (2007). Assessment of caregivers’ willingness to participate in an intervention research study. Res Nurs Health.

[70] Suttanon P, Hill KD, Said CM, Byrne KN, Dodd KJ, Suttanon P, Hill KD, Said CM, Byrne KN, Dodd KJ (2012). Factors influencing commencement and adherence to a home-based balance exercise program for reducing risk of falls: perceptions of people with Alzheimer’s disease and their caregivers. Int Psychogeriatr.

[71] Dow B, Moore K, Russel M, Ames D, Cyarto E, Haines T, Hill K, Lautenschlager N, Mackenzie L, Williams S, Loi S (2013). Improving mood through physical activity for carers and care recipients (IMPACCT): protocol for a randomised trial. J Physiother.

[72] Comans TA, Currin ML, Brauer SG, Haines TP (2011). Factors associated with quality of life and caregiver strain amongst frail older adults referred to a community rehabilitation service: implications for service delivery. Disabil Rehabil.

[73] Cristancho-Lacroix V, Wrobel J, Cantegreil-Kallen I, Dub T, Rouquette A, Rigaud A-S (2015). A web-based psychoeducational program for informal caregivers of patients with Alzheimer’s disease: a pilot randomized controlled trial. J Med Internet Res.

[74] Zarit SH, Lee JE, Barrineau MJ, Whitlatch CJ, Femia EE (2013). Fidelity and acceptability of an adaptive intervention for caregivers: an exploratory study. Aging Ment Health.

[75] Shim B, Barroso J, Davis LL (2012). A comparative qualitative analysis of stories of spousal caregivers of people with dementia: negative, ambivalent, and positive experiences. Int J Nurs Stud.

[76] Lach HW, Chang Y (2007). Caregiver perspectives on safety in home dementia care. West J Nurs Res.

[77] Rosler A, von Renteln-Kruse W, Muhlhan C, Frilling B (2012). Treatment of dementia patients with fracture of the proximal femur in a specialized geriatric care unit compared to conventional geriatric care. Z Gerontol Geriatr.

[78] Deschodt M, Braes T, Broos P, Sermon A, Boonen S, Flamaing J, Milisen K (2011). Effect of an inpatient geriatric consultation team on functional outcome, mortality, institutionalization, and readmission rate in older adults with hip fracture: a controlled trial. J Am Geriatr Soc.

[79] Reuben DB, Ganz DA, Roth CP, McCreath HE, Ramirez KD, Wenger NS (2013). Effect of nurse practitioner comanagement on the care of geriatric conditions. J Am Geriatr Soc.

[80] Ganz DA, Koretz BK, Bail JK, McCreath HE, Wenger NS, Roth CP, Reuben DB (2010). Nurse practitioner comanagement for patients in an academic geriatric practice. Am J Manag Care.

[81] Lichtenstein BJ, Reuben DB, Karlamangla AS, Han W, Roth CP, Wenger NS (2015). Effect of physician delegation to other healthcare providers on the quality of Care for Geriatric Conditions. J Am Geriatr Soc.

[82] Schrijvers G, van Hoorn A, Huiskes N (2012). The care pathway: concepts and theories: an introduction. Int J Integr Care.

[83] Deneckere S, Euwema M, Van Herck P, Lodewijckx C, Panella M, Sermeus W, Vanhaecht K (2012). Care pathways lead to better teamwork: results of a systematic review. Soc Sci Med.

[84] Vanhaecht K, Panella M, van Zelm R, Sermeus W (2011). What about care pathways?.

[85] Sherrington C, Fairhall NJ, Wallbank GK, Tiedemann A, Michaleff ZA, Howard K, Clemson L, Hopewell S, Lamb SE. Exercise for preventing falls in older people living in the community. Cochrane Database Syst Rev. 2019. 10.1002/14651858.CD012424.pub2 PMC6360922 30703272

[86] National Institute for Health and Care Excellence. Clinical practice guideline for the assessment and prevention of falls in older people. London; 2004. https://www.nice.org.uk/guidance/cg161. 31 August 2017

[87] Close JCT, Wesson J, Sherrington C, Hill KD, Kurrle S, Lord SR, Brodaty H, Howard K, Gitlin LN, O'Rourke SD, Clemson L (2014). Can a tailored exercise and home hazard reduction program reduce the rate of falls in community dwelling older people with cognitive impairment: protocol paper for the i-FOCIS randomised controlled trial. BMC Geriatr.

[88] Harwood RH, van der Wardt V, Goldberg SE, Kearney F, Logan P, Hood-Moore V, Booth V, Hancox JE, Masud T, Hoare Z (2018). A development study and randomised feasibility trial of a tailored intervention to improve activity and reduce falls in older adults with mild cognitive impairment and mild dementia. Pilot Feasibility Stud.

[89] Feldt KS (2000). The checklist of nonverbal pain indicators (CNPI). Pain Manag Nurs.

[90] Podsiadlo D, Richardson S (1991). The timed “up & go”: a test of basic functional mobility for frail elderly persons. J Am Geriatr Soc.

[91] Kiresuk TJ, Sherman RE (1968). Goal attainment scaling: a general method for evaluating comprehensive community mental health programs. Community Ment Health J.

[92] Kampe K, Kohler M, Albrecht D, Becker C, Hautzinger M, Lindemann U, Pfeiffer K (2017). Hip and pelvic fracture patients with fear of falling: development and description of the “step by step” treatment protocol. Clin Rehabil.

[93] Alzheimer’s Association (2017). Take care of yourself: how to recognize and manage caregiver stress.

[94] Alzheimer’s Society (2016). Factsheet 523LP: carers - looking after yourself.

