# Supplementary material for: Developing an Intervention for Fall-Related Injuries in Dementia (DIFRID): an integrated, mixed-methods approach
Source: BMC Geriatr. 2019 Feb 28;19:57. doi: 10.1186/s12877-019-1066-6 (PMC6394022; doi:10.1186/s12877-019-1066-6)
Supplement: Supplementary file 1 — Professional participants. Table showing numbers of participants from various health and social care professions in each stage of the project. (DOCX 12 kb) [file 12877_2019_1066_MOESM1_ESM.docx]

|  | **Initial qualitative interviews and focus groups** | **Consensus panel** | **Stakeholder interviews and focus groups following Delphi surveys** |
| --- | --- | --- | --- |
| Medics | 9 | 9 | 0 |
| Nursing | 21 | 2 | 1 |
| Professionals allied to medicine | 19 | 7 | 5 |
| Social care | 16 | 2 | 5 |
| 3^rd^ sector | 11 | 1 | 1 |
| Care home practitioner | 0 | 0 | 2 |
| Emergency services | 3 | 0 | 1 |
| Commissioners | 2 | 0 | 0 |
| Non-clinical academics | 0 | 3 | 0 |
| **Total** | **81** | **24** | **15** |
