# Supplementary material for: Developing an Intervention for Fall-Related Injuries in Dementia (DIFRID): an integrated, mixed-methods approach
Source: BMC Geriatr. 2019 Feb 28;19:57. doi: 10.1186/s12877-019-1066-6 (PMC6394022; doi:10.1186/s12877-019-1066-6)
Supplement: Supplementary file 2 — Comprehensive search strategy. Medline literature search strategy for the phase 1 comprehensive search. (DOCX 13 kb) [file 12877_2019_1066_MOESM2_ESM.docx]

Note: The Medline literature search strategy is provided below. This strategy was translated as necessary for each of the resources searched. This is one example of the initial broad search strategy. Parallel research strategies were created for other databases; the one shown below was for Medline (OVID).

### Example comprehensive search strategy

| 1. exp dementia/ |
| --- |
| 2. exp Supranuclear Palsy, Progressive/ or exp Hydrocephalus, Normal Pressure/ |
| 3. (Dementia? or Amentia? or Alzheimer*).ti,ab,hw,kw. |
| 4. ((Creutzfeldt-Jakob or huntington? or kluver-bucy or lewy-bod* or (lewy adj2 bod*)) adj3 (Syndrome or disease or disorder or dementia?)).ti,ab,hw,kw. |
| 5. ((normal adj2 hydrocephalus) or (supranuclear adj1 palsy) ((picks adj1 (disorder or disease)).ti,ab,hw,kw. |
| 6. or/1-5 |
| 7. ((Accidental* adj3 Fall?) or Falls or Fall-related or Fracture? or ((bone? or hip or femur or tibia or arm?) adj3 broken)).mp. |
| 8. (fall* adj3 injur*).mp. |
| 9. exp fractures, bone/ |
| 10. accidental falls/ |
| 11. or/7-10 |
| 12. exp accident prevention/ |
| 13. (preventi* or prevent).mp. |
| 14. intervention?.mp. |
| 15. exp Rehabilitation/ |
| 16. rehabilitat*.mp. |
| 17. exp Nutrition Therapy/ |
| 18. ((nutrition* or ergonomic or exercise or occupational or physical) adj3 (support* or therap*)).mp. |
| 19. physiotherap*.mp. |
| 20. (improv* adj5 (outcome? or care)).mp. |
| 21. management.mp. |
| 22. ((psycho* or physical* or mobility) adj5 (outcome? or improv*)).mp. |
| 23. (decreas* adj2 risk?).mp. |
| 24. ((improv* or increas*) adj5 (social* or participation or independence or activit* or well?being or QOL or (quality adj2 life))).mp. |
| 25. exp Activities of Daily Living/ |
| 26. ((multifactorial or multicomponent or multidisciplinary) adj3 (team? or assessment or intervention?)).mp. |
| 27. recovery.mp. |
| 28. HRQoL.mp. |
| 29. or/12-28 |
| 30. 6 and 11 and 29 |
