# Supplementary material for: Developing an Intervention for Fall-Related Injuries in Dementia (DIFRID): an integrated, mixed-methods approach
Source: BMC Geriatr. 2019 Feb 28;19:57. doi: 10.1186/s12877-019-1066-6 (PMC6394022; doi:10.1186/s12877-019-1066-6)
Supplement: Supplementary file 3 — Targeted search strategy. Example Medline literature search strategy for the phase 2 targeted searches. (DOCX 12 kb) [file 12877_2019_1066_MOESM3_ESM.docx]

### Example targeted search strategy

#### CMOc 1

The following search strategy was conducted to identify studies providing evidence for a connection between pain relief and rehabilitation outcomes. No such studies were found, only those providing evidence for a link between pain relief and aggressive/challenging behaviour, and studies reporting methods of assessing pain.

|  | Search strategy | Records | Notes |
| --- | --- | --- | --- |
| #9 | MM Pain AND (MM Dementia OR MM Aged+) AND MM Rehabilitation | 0 |  |
| #10 | (MM Dementia OR MM Aged+) AND MM Rehabilitation | 13 |  |
| #11 | (MM Dementia OR MM Aged+) AND TI (pain N2 (relie* or medic* or manag* or assess*)) OR AB (pain N2 (relie* or medic* or manag* or assess*)) AND MM Rehabilitation | 6 | Using keywords instead of thesaurus heading for Pain |
| #12 | (MM Dementia OR MM Aged+) AND TI (pain N2 (relie* or medic* or manag* or assess*)) OR AB (pain N2 (relie* or medic* or manag* or assess*)) AND TI (outcome* or benefit* or effect* or recover*) OR AB (outcome* or benefit* or effect* or recover*) | 73 | Using some keyword synonyms instead of thesaurus heading for Rehabilitation |
| #13 | (MM Dementia OR MM Aged+) AND MM Rehabilitation+ AND MM Pain+/pc | 2 | Using ‘explode’ for Rehabilitation; using ‘prevention & control’ subheading for Pain |
| #14 | MM Pain+/pc AND MH Dementia/rh | 0 | Using ‘rehabilitation’ subheading for Dementia |
