# Supplementary material for: Developing an Intervention for Fall-Related Injuries in Dementia (DIFRID): an integrated, mixed-methods approach
Source: BMC Geriatr. 2019 Feb 28;19:57. doi: 10.1186/s12877-019-1066-6 (PMC6394022; doi:10.1186/s12877-019-1066-6)
Supplement: Supplementary file 4 — Delphi survey. Expanded description of rationale and methodology for the Delphi survey. (DOCX 13 kb) [file 12877_2019_1066_MOESM4_ESM.docx]

| **Purpose of study** |
| --- |
| To determine the feasibility of developing and implementing a new intervention for people with dementia with fall-related injuries. |
| **Rationale for Delphi** |
| Stakeholders to assist in synthesising evidence collected in the previous parts of the study and make recommendations for the design of the new intervention. |
| **Selection of expert panel members** |
| (1) representation of a range of stakeholder groups identified to be important to the care of PWD who fall, including geriatricians, old age psychiatrists, emergency department consultant, occupational therapist, physiotherapist, general nurse, mental health nurse, commissioner, and social services  (2) in contact with older PWD who fall and/ or  (3) relevant academic expertise  (4) able to travel to the research site for face to face meetings |
| **Description of methods** |
| Following the first consensus panel meeting, a series of statements were identified and sent to panel members via an online survey tool. A second round of the survey was conducted which included the results of the first round, giving members the opportunity to revise their responses.  Only the independent moderator (BE) had access to non-anonymised data. |
| **Definition of consensus** |
| Two-thirds agreement |
| **Pilot test of instruments** |
| The questions were discussed at a meeting of the Programme Management Group and refined before the survey was administered. |
| **Data analysis** |
| Statistical analysis software was used to describe the proportions agreeing or disagreeing with each statement |
| **Justification of number of rounds** |
| Two rounds was chosen to give participants the chance to adjust their responses based on the results of the initial round. Any remaining non-consensus was resolved at the second panel meeting. |
| **Limitations** |
| While a response rate of 58% was achieved for the consensus surveys, not all panel members attended the consensus meetings. The panel also did not include PPI representatives. Methods seemed less accessible for social care professionals, as evidenced by difficulty recruiting panel members and engaging them in the Delphi process. These factors may have implications for the results. |
