# Supplementary material for: Developing an Intervention for Fall-Related Injuries in Dementia (DIFRID): an integrated, mixed-methods approach
Source: BMC Geriatr. 2019 Feb 28;19:57. doi: 10.1186/s12877-019-1066-6 (PMC6394022; doi:10.1186/s12877-019-1066-6)
Supplement: Supplementary file 5 — Consensus statements. Full list of statements provided to the consensus panel along with their outcomes. (DOCX 16 kb) [file 12877_2019_1066_MOESM5_ESM.docx]

| **Statement** | **Outcome** | **Round** | **Percentage** | **Final Selection** |
| --- | --- | --- | --- | --- |
| **Feasibility, design and inclusion criteria of the study** | | | | |
| The brief requires us to design a complex intervention. | Agreed | 1 | 92.9 | As statement |
| Patients with non-injurious falls should be eligible for the intervention | Agreed | 1 | 100.0 | As statement |
| Fallers with an acute medical illness causing their fall, e.g. pneumonia or stroke, are included | No consensus | 2 | 46.2 | Include |
| Fallers should be recruited either within 1 week of the fall or 1 month of the fall | No consensus | 2 | 53.8, 46.2 | 1 month |
| A feasible and useful sample size would be (% given for range of choices up to 30 participants) | No consensus | 1 | 64.3 | up to 30 participants |
| The number of sites included should be 3 sites. | Agreed | 2 | 76.9 | 3 sites |
| It is feasible to recruit to WP4 | Agreed | 2 | 100.0 | As statement |
| **Setting of the study** | | | | |
| It would be useful to recruit participants presenting with a fall in the ED | Agreed | 1 | 92.9 | As statement |
| It would be useful to recruit participants presenting with a fall to paramedics if single ambulance stations are targeted | Agreed | 1 | 92.9 | As statement |
| It would be useful to recruit participants presenting with a fall in the primary care setting | Agreed | 1 | 85.8 | As statement |
| *If we are recruiting participants who have had a fall within the last week, it would be useful for GPs to write to all patients on their QOF dementia register* | *No consensus* | *2* | *30.8* | *Rejected as we will not be recruiting patients up to one week after a fall* |
| If we are recruiting participants who have had a fall within the last month, it would be useful for GPs to write to all patients on their QOF dementia register | Agreed | 2 | 84.6 | As statement |
| It would be useful to recruit participants in another setting. | Agreed | 1 | 78.6 | As statement |
| Mean priorities for alternative settings: | | | | |
| Community services e.g. multidisciplinary outreach teams |  |  | 3.1 | Include |
| *Domiciliary physiotherapy* |  |  | *4.6* | *Exclude* |
| Supported discharge teams |  |  | 3.4 | Include |
| Telecare services |  |  | 3.4 | Include |
| *Social services re-enablement teams* |  |  | *4.4* | *Exclude* |
| *Memory clinics* |  |  | *4.4* | *Exclude* |
| *Dementia cafes* |  |  | *5.5* | *Exclude* |
| *Social media* |  |  | *7.2* | *Exclude* |
| The intervention should primarily take place in the patient's home | Agreed | 1 | 85.7 | As statement |
| The setting of the intervention should make use of existing pathways only when referral from the team deems it would be useful for the individual | Agreed | 1 | 85.7 | As statement |
| **Content of the intervention (staff)** | | | | |
| A Physiotherapist should be routinely involved | Agreed | 1 | 71.4 | As statement |
| An Occupational therapist should be routinely involved | Agreed | 1 | 71.4 | As statement |
| A Geriatrician should be routinely involved via multidisciplinary team meeting and available for face to face consultation if required | No consensus | 2 | 61.5 | As statement |
| A Rehabilitation support worker should be routinely involved | Agreed | 1 | 71.4 | As statement |
| A Registered general nurse should be routinely involved via multidisciplinary team meeting and available for face to face consultation if required | No consensus | 2 | 61.5 | Included only where MDTs already include a nurse |
| A Community psychiatric nurse should be available on referral | Agreed | 1 | 71.4 | As statement |
| A Social worker should be available on referral | Agreed | 1 | 71.4 | As statement |
| Re-enablement workers should be available on referral | Agreed | 1 | 71.4 | As statement |
| An Old Age Psychiatrist should be available on referral | Agreed | 2 | 84.6 | As statement |
| A Podiatrist should be available on referral | Agreed | 2 | 92.3 | As statement |
| **Content of the intervention (assessment)** | | | | |
| Assessment should involve multiple sources of information including information from carers | Agreed | 1 | 100.0 | As statement |
| Assessment should include direct observation | Agreed | 1 | 100.0 | As statement |
| Formal assessments of gait and balance should be carried out by the Timed Up and Go test | No consensus | 2 | 61.5 | As statement |
| A home hazard assessment should include a walk around the house to determine where actual falls have occurred and negotiate how these might be reduced | Agreed | 1 | 92.9 | As statement |
| An assessment of comorbidities is required | Agreed | 1 | 100.0 | As statement |
| An osteoporosis risk assessment is required | Agreed | 1 | 92.9 | As statement |
| A vision assessment is required | Agreed | 1 | 100.0 | As statement |
| A medication review is required | Agreed | 1 | 100.0 | As statement |
| All patients require attendance for a lying and standing BP | No consensus | 2 | 53.8 | To be carried out by therapist in the patient's home |
| A continence assessment is required | Agreed | 1 | 78.6 | As statement |
| An assessment of challenging behaviour is required | Agreed | 1 | 92.9 | As statement |
| Tools which assess non-verbal signs of pain should be used | Agreed | 1 | 92.9 | As statement |
| A multidisciplinary team meeting should be available if needed | Agreed | 1 | 92.9 | As statement |
| Carer stress should be routinely assessed | Agreed | 1 | 92.9 | As statement |
| **Content of the intervention (methodology and quantity)** | | | | |
| Interventions should be based on goals set by the patient and carer | Agreed | 1 | 85.7 | As statement |
| Therapists should work with service users to minimise the risk of falling, as this may improve confidence and enable realistic risk taking. | Agreed | 1 | 100.0 | As statement |
| Therapists should facilitate caregivers, family and friends to adopt a positive approach to risk | Agreed | 1 | 100.0 | As statement |
| Exercise interventions should be informed by evidence based formats such as the Otago programme but tailored to the circumstances of people with dementia and embedded in their daily life | Agreed | 2 | 69.2 | As statement |
| The total number of physiotherapy sessions available in the first 3 months (including sessions delivered by a support worker) should be 16, 20 or 24 | No consensus | 2 | 30.8, 38.5, 30.8 | 20 sessions: Twice weekly (weeks 0-8) tapering to once weekly (weeks 9-12) |
| The total number of occupational therapy sessions available in the first 3 months should be 3-4 | No consensus | 2 | 61.5 | 4 |
| Therapists should offer service users information on assistive devices and facilitate delivery | Agreed | 1 | 100.0 | As statement |
| Therapists should help the service user and caregiver to develop a meaningful programme of activities | Agreed | 1 | 100.0 | As statement |
| Therapists should undertake observed activities with the service user to facilitate new learning | Agreed | 1 | 92.9 | As statement |
| Intervention staff should be able to provide basic carer education & support, referring to other agencies as needed | Agreed | 2 | 76.9 | As statement |
| **Staff training** | | | | |
| Tier 2 training is required for intervention staff | Agreed | 2 | 84.6 | As statement |
| Training needs to include how to tailor an intervention for people with dementia | Agreed | 1 | 100.0 | As statement |
| Training needs to include advice on how to engage and motivate people with dementia | Agreed | 1 | 100.0 | As statement |
| Training should include on the job role modelling | Agreed | 1 | 100.0 | As statement |
| **Outcome measures for the intervention** | | | | |
| The primary outcome measure be a numerical measure of falls | Agreed | 2 | 76.9 | As statement |
| Secondary outcomes should include health related quality of life measure | Agreed | 1 | 100.0 | As statement |
| The best health related quality of life measure would be Quality of life in Alzheimer’s disease (QOL-AD) | Agreed | 2 | 69.2 | As statement |
| Secondary outcomes should include activities of daily living measure | Agreed | 1 | 92.9 | As statement |
| The best activities of daily living measure would be Disability Assessment for Dementia (DAD) | Agreed | 2 | 84.6 | As statement |
| Secondary outcomes should include carer burden measure | Agreed | 1 | 92.9 | As statement |
| The best carer burden measure would be Zarit Burden interview | Agreed | 2 | 69.2 | As statement |
| Secondary outcomes should include psychological consequences of falling measure | Agreed | 1 | 85.7 | As statement |
| The best psychological consequence measure e.g. fear of falling would be the Modified Falls Efficacy scale | Agreed | 1 | 71.4 | As statement |
| Secondary outcomes should include physical activity measure | No consensus | 1 | 64.2 | As statement |
| The best physical activity measure would be a wearable physical activity monitor | Agreed | 1 | 78.6 | As statement |
| Secondary outcomes should include Strength and balance measure | No consensus | 1 | 57.1 | TUAG as in initial assessement |
| Secondary outcomes should include goal setting or performance measure | No consensus | 1 | 35.7 | As statement |
| The best goal setting or performance measure would be Goal Attainment scaling | Agreed | 2 | 84.6 | As statement |
| *The best carer quality of life measure would be EQ-5D- 5L* | *No consensus* | *1* | *57.1* | *Exclude* |
| The most popular Carer quality of life measure was EQ-5D-5L, but it was suggested that a measure of carer burden would be sufficient. | No consensus | 2 | 53.8 | As statement |
| **Prioritise the remaining domains where consensus was not achieved (1 highest - 4 lowest)** | | | | |
| Goal setting measure |  | 2 | 2.0 | Include |
| Physical activity measure |  | 2 | 2.5 | Include |
| Strength and balance measure |  | 2 | 2.5 | Include |
| *Carer quality of life* |  | *2* | *3.0* | *Exclude* |
