# Supplementary material for: Developing an Intervention for Fall-Related Injuries in Dementia (DIFRID): an integrated, mixed-methods approach
Source: BMC Geriatr. 2019 Feb 28;19:57. doi: 10.1186/s12877-019-1066-6 (PMC6394022; doi:10.1186/s12877-019-1066-6)
Supplement: Supplementary file 6 — Final DIFRID intervention. A description of the final intervention using the TIDIeR framework. (DOCX 16 kb) [file 12877_2019_1066_MOESM6_ESM.docx]

| **Brief name**  DIFRID: Developing an intervention for fall-related injuries in dementia |
| --- |
| **Why**  People with dementia (PWD) are more likely to fall, have more difficulty recovering and stay in hospital for longer than people without dementia. However, they are often excluded from multifactorial interventions aimed at falls prevention and/or recovery. The results of the initial exploratory part of our study suggest it is both possible and desirable to develop an intervention to improve the outcome of fall-related injuries in PWD. The underlying principles of the new intervention are: ensuring that the circumstances of rehabilitation are optimised for PWD; compensating for the reduced ability of PWD to self-manage; equipping the workforce with the necessary skills and information to care for this patient group. |
| **What - Materials**   - A comprehensive Assessment and Intervention Document will be provided to therapists to use in assessing each participant and planning intervention sessions. - A Study Manual will be provided to all staff delivering the intervention, in order to clarify procedures. - Training slides will be used in delivering training to staff. All the information contained on these will be included in the manual. - A blood pressure monitor will be provided to the intervention team at each site. - Additional materials (e.g. exercise sheets) may be provided to participants at the discretion of individual therapists. Yellow paper will be provided to therapists to make these dementia friendly. - Carer education leaflets will also be provided to therapists for distribution as required. - A Patient Diary will be provided to participants; activities are recorded within for reference as well as study data collection.   **What - Procedures**  Each participant will receive one physiotherapy and one occupational therapy assessment in their home. As part of the assessment, the participant’s likes, dislikes and personal goals will be recorded. An assessment of carer need is also included. Following these assessments a multidisciplinary team meeting will be held to decide the most appropriate activity programme to achieve the participant’s goals, as well as to identify and carry out appropriate referrals to a geriatrician or to other services (for example podiatry or carer support services). The participant will then receive intervention sessions at home in order to support their programme of activities. During each session, their progress will be recorded and their programme of activities adjusted as required. At the end of the intervention, the participant’s ongoing needs will be discussed with them and referrals to other services (such as strength and balance classes) are made as appropriate. |
| **Who provided**  Assessments will be carried out by physiotherapists and occupational therapists.  Intervention sessions may be carried out by physiotherapists, occupational therapists, or therapy assistants/reablement support workers.  All staff involved in delivering the intervention will have completed study-specific training as part of the intervention, which includes information on working with people with dementia. This training is provided by the intervention team, consisting of a physiotherapist and an occupational therapist. |
| **How**  The intervention will be delivered individually and face to face. A family carer should be present for the assessment visits but is not required to be present for the intervention sessions. Multidisciplinary team meetings may be held face to face or remotely, according to the team’s preferred practice. |
| **Where**  Assessments and intervention sessions will be delivered in the participant’s own home, or another appropriate location depending on patient goals and activity plans.  Staff training and multidisciplinary team meetings may be held in a location that is most convenient for the team. |
| **When and How Much**  The intervention is designed to be flexible according to individual needs. Each participant may have up to four sessions with a physiotherapist, up to four sessions with an occupational therapist and up to 14 sessions with a support worker over a period of 12 weeks. Each intervention session will last an average of one-hour, but this can be adjusted according to need and the nature of the programme of activities. Review sessions are scheduled at six weeks and 12 weeks.  The staff training will be delivered in one half-day session. |
| **Tailoring**  The intervention is designed to be tailored to each participant. After assessment, the programme of activities and number/frequency of sessions will be decided according to the needs of the individual. This will take place at the multidisciplinary meeting and may also be adjusted during the course of the intervention, for example if the participant is progressing at a different pace than expected with the goals set. |
